# Retraction: Apelin Receptor (APJ) Expression during Cardiopulmonary Bypass in Children Undergoing Surgical Repair

**DOI:** 10.1371/journal.pone.0202136

**Published:** 2018-08-06

**Authors:** The PLOS ONE Editors

The *PLOS ONE* Editors retract this article [1] in light of concerns that were raised about Figure 1A. Specifically, it was noted that all 13 lanes in the HSP70 panel appear similar to one another and to the band for patient 15C in the APJ receptor blot.

First author SW notified the journal that the original data underlying these figure panels are no longer available.

The University of Glasgow investigated these concerns and recommended retraction owing to signs of data manipulation and falsification. In line with the institution’s communication, the *PLOS ONE* Editors retract this article, as the concerns raised call into question the integrity of the data and the validity of the article’s results and conclusions.

SW, MHDD did not agree with the retraction. ADL, FL did not respond.
